# Troxler fading, eye movements, and retinal ganglion cell properties

**DOI:** 10.1068/i0679sas

**Published:** 2014-11-28

**Authors:** Romain Bachy, Qasim Zaidi

**Affiliations:** Graduate Center for Vision Research, SUNY College of Optometry, New York, USA; e-mail: rbachy@sunyopt.edu; Graduate Center for Vision Research, SUNY College of Optometry, New York, USA; e-mail: qz@sunyopt.edu

**Keywords:** eye movement, Troxler fading, adaptation, after-image, retinal ganglion cells

## Abstract

We present four movies demonstrating the effect of flicker and blur on the magnitude and speed of adaptation for foveal and peripheral vision along the three color axes that isolate retinal ganglion cells projecting to magno, parvo, and konio layers of the LGN. The demonstrations support the eye movement hypothesis for Troxler fading for brightness and color, and demonstrate the effects of flicker and blur on adaptation of each class of retinal ganglion cells.

Troxler (1804) showed that fixated stimuli fade faster in peripheral than central vision. Clarke and Belcher (1962) attributed the effect to miniature fixational eye-movements having a greater refreshing effect on retinal cells at the edges of the stimulus in central versus peripheral vision, because central receptive fields are smaller than peripheral receptive fields. Bachy and Zaidi (2014) added blur and flicker to the color after-images method introduced by Zaidi, Ennis, Cao, and Lee (2012) to support the eye-movement-based hypothesis, buttressed by electrophysiological results showing no effect of eccentricity between 2 and 12 degrees on time-constants of retinal ganglion cell adaptation. In this paper, we present four movies demonstrating the effect of flicker and blur on adaptation along the three cardinal color axes that isolate the responses of the three types of ganglion cells: Δ(L + M + S) gray-level modulation isolates cells that project to magno layers in the LGN, Δ(L − M) equiluminant reddish–greenish modulation isolates cells that project to the parvo layers, and Δ(S) equiluminant bluish–yellowish modulation isolates cells that project to konio cells (Sun, Smithson, Zaidi, & Lee, 2006).

Movie 1 (see supplementary online material) illustrates color after-effects induced by a time-varying procedure taking 45 s applied to a classic after-image demonstration (Sadowski, 2006). Beginning from a gray-scale image, negative colors of a picture are slowly modulated up to a maximum of contrast then slowly back to the initial gray with a half-sinusoid time-course (32 sec). For a fixating observer, while the color contrast of the picture slowly goes back to gray, natural colors slowly appear on the picture for a while, and then disappear to go back to the initial gray.

In Movie 2 (see supplementary online material), a time-varying stimulus modulates along the light–dark achromatic axis. By judging which stimulus fades first to gray before evoking a negative after-image, an observer can directly compare the speed of peripheral adaptation against central adaptation, i.e., the Troxler effect, under three conditions. The three conditions of the movie demonstrate the effects of central blur and peripheral flicker on the speed of adaptation: First, the three stimuli have same shape and temporal properties, the classic Troxler effect is observed with peripheral stimuli disappearing faster than the central one; second, we add blurred edges to the central stimulus in order to decrease the effect of eye movements, the central stimulus disappears significantly faster; third, we add intermittent flicker to the peripheral stimuli in order to simulate the effect of eye movements that intermittently shift the positions of receptive fields between the background and the stimulus (for details see Bachy & Zaidi, 2014), but this does not have an effect different from condition 2.

In Movie 3 (see supplementary online material), a time-varying stimulus modulates along the reddish–greenish axis in the three conditions demonstrating the effects of central blur and peripheral flicker on the speed of adaptation of the parvo-cell pathway: First, the three stimuli have same shape and temporal properties, a chromatic Troxler effect is observed with peripheral stimuli disappearing faster than the central one; second, blurred edges of the central stimulus decrease the effect of eye movements, but unlike for the achromatic brightness stimuli, this does not change the relative speeds of central and peripheral adaptation; third, intermittent flicker of the peripheral stimuli makes the peripheral stimuli disappear significantly slower.

In Movie 4 (see supplementary online material), a time-varying stimulus modulates along the yellowish–bluish axis of color space that isolates the adaptation properties of the konio-cell pathway. For the three conditions, similar results to Movie 3 are observed.

These demonstrations support the eye movement hypothesis for Troxler fading for both color and brightness by showing directly the difference of adaptation time-course with eccentricity. However, they also show that the effect is mediated by spatial and temporal response properties of ganglion cells: Magno cells are responsive to much higher spatial frequencies than are parvo or konio cells. Blurring the edge then has a bigger effect on adaptation of magno cells than on parvo or konio cell, and this was reflected in the psychophysical results (Bachy & Zaidi, 2014). The flicker creates transient responses in parvo and konio cells, and these responses are similar to the eye-movement-caused transient responses in magno cells (Ennis, Cao, Lee, & Zaidi, 2014). As a result, peripheral adaptation slows down for the chromatic axes to be roughly similar to the achromatic peripheral adaptation (Bachy & Zaidi, 2014). Consequently, ganglion cell properties must be taken into account for the Troxler effect, not just the increasing size of receptive fields as a function of eccentricity.
